# D-CAPS: an efficient CRISPR-Cas9-based phage defense system for *E*. *coli*: A novel strategy for *E*. *coli* to resist T7 based on CRISPR-Cas9

**DOI:** 10.3724/abbs.2024208

**Published:** 2025-04-28

**Authors:** Mingjun Sun, Jie Gao, Hongjie Tang, Hengyi Wang, Liyan Zhou, Chuan Song, Yongqiang Tian, Qi Li

**Affiliations:** 1 College of Life Sciences Sichuan Normal University Chengdu 610101 China; 2 Luzhou Laojiao Co. Ltd. Luzhou 646000 China; 3 National Engineering Research Center of Solid-State Brewing Luzhou 646000 China; 4 Key Laboratory of Leather Chemistry and Engineering (Sichuan University) Ministry of Education Chengdu 610065 China; 5 College of Biomass Science and Engineering Sichuan University Chengdu 610065 China

**Keywords:** Keywords: bacteriophage contamination, CRISPR-Cas9, *E*. *coli*, T7 phage

## Abstract

*Escherichia coli* is widely used in industrial chemical synthesis but faces significant challenges due to bacteriophage contamination, which reduces product quality and yield. Therefore, developing an efficient antiphage system is essential. In this study, we develop a CRISPR-Cas9-based antiphage system (CAPS) targeting essential genes of the T7 phage (gene *5* and gene *19*) with single gRNAs transformed into MG1655 strains expressing Cas9. While CAPS provides limited resistance, with plating efficiencies ranging from 10 ^–5^ to 10 ^–1^, further optimization is needed. To enhance efficacy, we design a double-site-targeting CRISPR-Cas9-based antiphage system (D-CAPS). D-CAPS demonstrates complete resistance, with no plaques observed even at a high multiplicity of infection (MOI of 2), and growth curve analysis reveals that antiphage *E*. *coli* strains grow normally, similar to the wild-type strain, even at a high multiplicity of infection. Furthermore, D-CAPS is effective against BL21(DE3) strains, showing strong resistance and demonstrating its versatility across different *E*. *coli* strains. Protein expression analysis via green fluorescent protein confirms that *E*. *coli* carrying D-CAPS could maintain normal protein expression levels even in the presence of phages, comparable to wild-type strains. Overall, D-CAPS offers a robust and versatile approach to enhancing *E*. *coli* resistance to phages, providing a practical solution for protecting industrial *E*. *coli* strains and improving fermentation processes.

## Introduction

*Escherichia coli* strains are the most commonly used hosts in industrial production and are widely utilized in the synthesis of various chemicals, including organic acids, alcohols, amino acids, fatty acids, alkanes, *etc*. [ 1– 9]. Notably, *E*. *coli* strains such as MG1655 from the K-12 series and BL21 (DE3) from the B series have become important chassis strains in synthetic biology [ 10– 12]. However, in actual industrial production, *E*. *coli* faces the risk of contamination by bacteriophages [13]. Phages can rapidly kill host bacteria, accumulate at very high concentrations, and spread throughout production facilities. This contamination can lead to a decline in fermentation product quality, reduced production capacity, and economic losses [ 14, 15]. Therefore, addressing the issue of phage contamination in fermentation processes is urgent. Various industries have implemented methods to reduce contamination by bacteriophages during fermentation, such as raw material treatment, strain rotation, process changes, plant design adjustments, and extensive cleaning and hygiene practices [ 16, 17]. However, these measures undoubtedly increase the operating costs of facilities.

*E*. *coli* possesses several endogenous mechanisms to resist phage infection, including surface receptor mutation, the restriction-modification (R-M) system, the clustered regularly interspaced short palindromic repeats (CRISPR)-CRISPR-associated protein (Cas) system, the abortive infection system, and the Thoeris system [ 18– 22]. These mechanisms help *E*. *coli* survive phage infection or prevent phage contamination from spreading in the environment. Although *E*. *coli* has evolved a wide variety of antiphage strategies through its long-term arms race with bacteriophages, bacteriophages have concurrently evolved various countermeasures to evade these defense systems [23]. Consequently, the endogenous defense mechanisms of *E*. *coli* offer limited protection during actual phage infections [ 22, 24]. Thus, it is necessary to develop more effective measures to defend against bacteriophage infection.

To achieve the above goals, introducing exogenous antiphage strategies into chassis strains has become a popular approach. Methods such as incorporating R-M systems, Abi systems, and antisense RNA systems have achieved significant results [ 25– 27]. However, these methods commonly face problems such as the inability to withstand high concentrations of phage infections, reliance on complex strain rotation during practical application, or limited bacterial growth capabilities. With the rise of CRISPR-Cas technology, researchers have attempted to express the CRISPR-Cas9 system in *E*. *coli* to achieve immunity against phages [28]. However, the system cannot resist T7 phage infections completely but only delays the lysis of the host, significantly impacting the growth of the host bacteria. Therefore, optimization of this method is urgently needed.

In this study, we constructed an efficient phage defense system based on CRISPR-Cas9 in *E*. *coli* to counter the T7 phage, a fast-lysing and industrially harmful bacteriophage. By targeting essential genes in the T7 phage, we found that a single-site targeting system provided partial resistance, while a double-site targeting system (D-CAPS) completely resisted T7 phage infection, allowing robust bacterial growth. This CRISPR-Cas9-based system provides a solution for mitigating T7 phage contamination in industrial fermentation processes and maintaining protein expression capacity in *E*. *coli*.

## Materials and Methods

### Bacterial strains and culture methods

*E*. *coli* strains DH5α, MG1655, and BL21(DE3) stored in our lab were selected and cultured at 37°C in LB media [0.5% (w/v) yeast extract, 1% (w/v) peptone, and 1% (w/v) NaCl]. The following antibiotics were added to the LB medium as needed: chloramphenicol (25 μg/mL), spectinomycin (100 μg/mL), kanamycin (50 μg/mL), and ampicillin (100 μg/mL). The strains were stored at ‒80°C in 25% glycerol. To recover the strains, the cultures were streaked on LB agar plates, and the isolated colonies were inoculated into LB broth. The T7 phage was propagated in the BL21(DE3) strain during its logarithmic growth phase. All strains used in this study are listed in Supplementary Table S1.

### Reagents and enzymes

The restriction endonucleases used in this study were purchased from Thermo Fisher Scientific (Waltham, USA). Phanta Flash Master Mix (2×) from Vazyme Biotechnology (Nanjing, China) and Es Taq MasterMix (2×) with dye from Jiangsu Cowin Biotech (Taizhou, China) were used for high-fidelity DNA amplification and colony PCR, respectively. Plasmid extraction kits were obtained from Tiangen Biotech (Beijing, China), and DNA purification reagents were obtained from TransGen Biotech (Beijing, China) and used following the manufacturers’ instructions. Plasmid assembly was performed via the ClonExpress One Step Cloning Kit from Vazyme Biotechnology.

### Plasmid construction

*E*. *coli* DH5α was used for plasmid maintenance and construction. All plasmids and primers used in this study are listed in Supplementary Tables S1 and S2. The pEcCas plasmid (Addgene number: 73227) originated from our previous research, and the pEcgRNA-X series plasmids (where X represents the targeted gene) were constructed according to our published methods [29].

### Plasmid transformation and determination of the EOP

The pEcCas and pEcgRNA-targetX plasmids were electroporated into *E*. *coli* strains as experimental strains, with the wild-type *E*. *coli* strains serving as control strains. For escape rate testing, 300 μL of logarithmic-phase experimental or control *E*. *coli* cells (10 ^8^ CFU/mL) were mixed with 5 mL of 0.8% agarose semisolid LB medium, which was then poured onto solid LB plates. Different dilutions of T7 phage in LB medium were spotted onto solidified plates (from stock solution to a 10 ^–8^ dilution). The plates were incubated overnight at 37°C. The plaque-forming units (PFUs) of the phage were counted, and the phage survival rate was calculated. The plaque counting method is as follows: among the plaque groups formed by different concentrations of phages, the group where the number of plaques is just countable is selected. The number of plaques for that group was calculated by multiplying the plaque count by the dilution factor of that group. Each experiment was repeated three times.

The measurement of EOP requires both the experimental strain and the control strain. It is calculated as the ratio of the number of plaques formed by the same quantity of phages on these different strains, specifically (PFU of experimental group phages on double-layer plates)/(PFU of control group phages on double-layer plates). Generally, the control strain should be the optimal host for the phage, and for the T7 phage, wild-type BL21(DE3) is typically used. Therefore, the higher the EOP of the experimental strain is, the closer it is to an ideal host, and the lower its resistance to the phage.

### Determination of the growth curve of *E*. *coli* infected with the T7 phage

Three microlitres of T7 phage stock solution stored in our lab (10 ^8^ PFU/μL) was added to 30 mL of *E*. *coli* culture in the logarithmic growth phase. The culture mixture was incubated in a shaking incubator at 37°C and 220 rpm. The OD _600_ values were measured with an ultraviolet spectrophotometer from Thermo Fisher Scientific (Waltham, USA) at 1 h, 2 h, 3 h, 4 h, 6 h, 8 h, and 12 h. The control group consisted of wild-type *E*. *coli*, while the blank control group had an equal volume of LB medium added instead of the T7 phage. Each experiment was repeated three times.

### Fluorescence expression assay

The plasmid pGFP stored in our lab, which expresses GFP, was transformed into each *E*. *coli* strain. After the logarithmic growth phase was reached, 3 μL of phage stock solution (10 ^8^ PFU/μL) was added. Simultaneously, the same amount of T7 phage was added to wild-type *E*. *coli* MG1655 containing the pGFP plasmid as the control group. Wild-type *E*. *coli* MG1655 containing the pGFP plasmid without T7 phage was used as a blank control. Cultures were incubated in a shaking incubator at 37°C and 220 rpm. The fluorescence intensity of each culture was observed at 1 h, 2 h, 3 h, 4 h, 6 h, 8 h, and 12 h.

### Statistical analysis

Statistical analyses were performed by a *t*-test using the IBM SPSS Statistics program (version 21) to determine the significant differences between the control and experimental groups. Graphs were prepared using Graphpad Prism (version 9). *P*  < 0.05 was considered to indicate significant difference.

## Results

### Designing CRISPR-Cas9 with gRNA targeting a single site on the T7 phage genome can enable *E*. *coli* MG1655 to defend against phage infection

**Figure 1:**
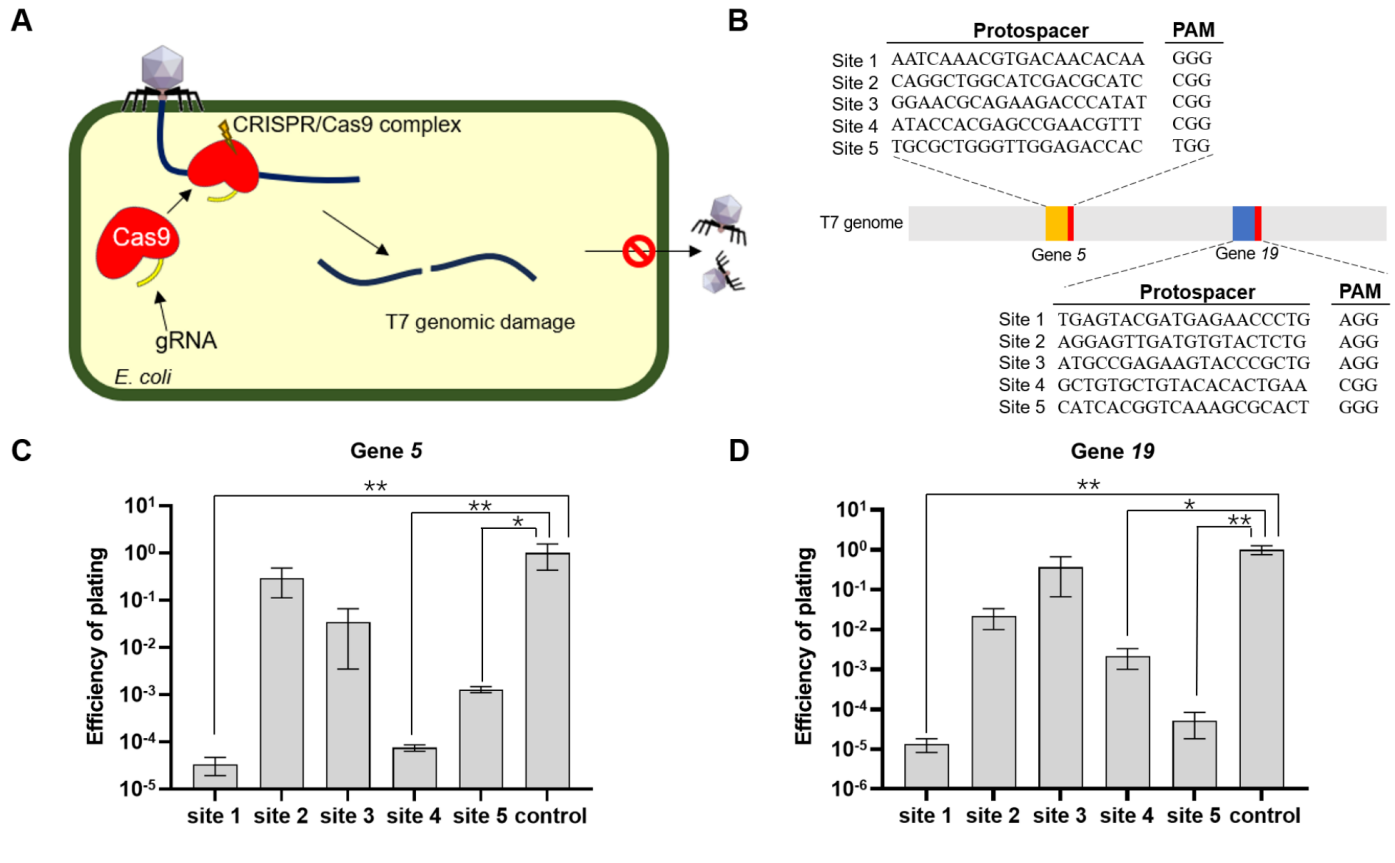
Figure 1 CAPS can enable MG1655 to defend against phage infection (A) Schematic representation of the function of CAPS. (B) T7 phage-targeted sites in this study. (C) MG1655, with CAPS targeting gene 5 of T7, provides defense against phage infection. (D) MG1655, with the CAPS-targeting gene 19 of T7, provides defense against phage infection. The “control” represents wild-type MG1655; *P < 0.05; **P < 0.01.

Theoretically, CRISPR-Cas9-mediated double-strand breaks (DSBs) in genomic DNA are lethal to bacteriophages, especially when breaks in essential genes occur ( Figure 1A). Therefore, to ensure efficient cleavage, essential genes *5* and *19* of the T7 bacteriophage, which are involved in genome replication and assembly, respectively, were selected, and five gRNAs for each gene were designed ( Figure 1B). The corresponding gRNA plasmids were subsequently constructed, creating a CRISPR-Cas9-based antiphage system (CAPS), which was subsequently transformed into *E*. *coli* MG1655, which constitutively expresses Cas9 via the plasmid pEcCas9. Next, these *E*. *coli* strains carrying pEcCas9 and the corresponding pEcgRNA plasmids were infected with T7 phage. The EOP of the T7 phage on these strains was calculated to evaluate the ability of the host to resist T7 phage infection. A lower EOP indicates a greater ability of the host to resist phage infection and stronger resistance to the T7 phage.

Across all the tested sites, the EOP of the T7 phage ranged from 10 ^–5^ to 10 ^–1^ ( Figure 1C,D). Plaques were always visible on the double-layer plates, indicating that some phages survived the cleavage of Cas9 at these sites through certain mechanisms ( Supplementary Figure S1). This observation was consistent with our previous studies [24]. On the basis of our earlier work, we speculate that the lower EOP sites may be resulted from synonymous mutations in the targeted genes of the T7 phage.

**Figure 2:**
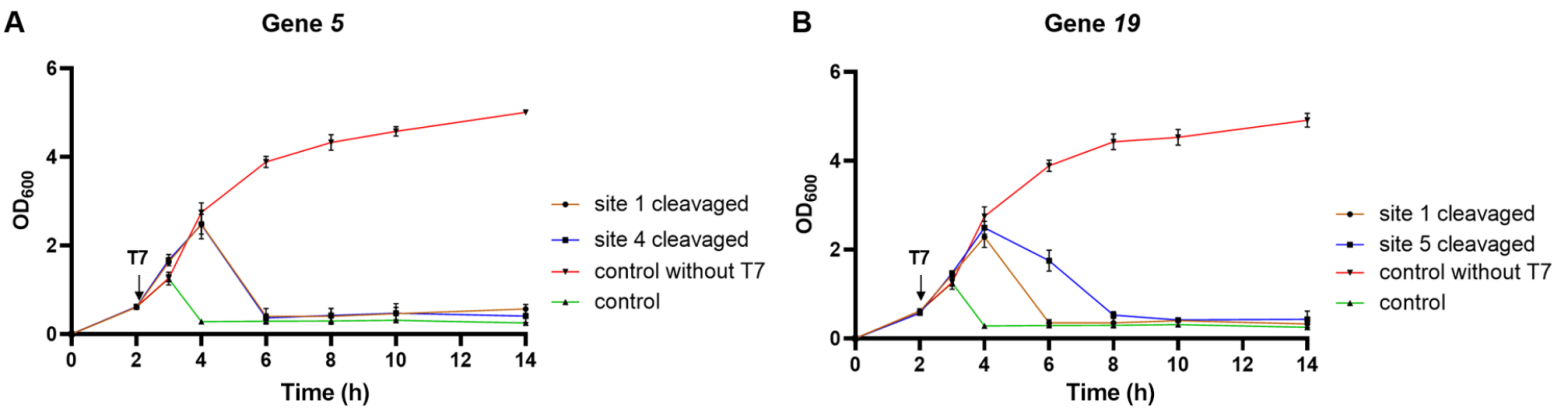
Figure 2 CAPS can enable MG1655 to defend against phage infection (A) Growth curve showing that MG1655, with CAPS targeting the T7 phage gene 5, can partially resist T7 infection at an MOI of 0.02. (B) Growth curve showing that MG1655, with CAPS targeting the T7 phage gene 19, can partially resist T7 infection at an MOI of 0.02. The “control” represents the wild-type MG1655 with T7.

Next, two sites with the lowest EOP in each gene were selected, including sites 1 and 4 for gene *5* and sites 1 and 5 for gene *19*. The growth curves of *E*. *coli* MG1655 strains targeting these sites were plotted after infection with the T7 phage at an MOI of 0.02. The results showed that these strains that target single essential gene sites of the T7 phage ultimately cannot completely resist T7 phage infection. Within 1–2 h of T7 phage addition, the growth of these strains was almost identical to that of the wild-type MG1655. The wild-type MG1655 were nearly completely lysed by the T7 phage. However, within 2–4 h, these anti-T7 strains targeting single genome sites of the T7 phage were still lysed by the T7 phage. By 6 h, almost all the strains were completely lysed ( Figure 2A,B). Interestingly, site 1-targeted *p19* offered better protection against T7 in the plating efficiency calculations but was less effective in the growth curve analysis (Figures 1D and 2B). We speculate that this may be due to differences in the cleavage efficiency of certain sites between solid and liquid media. These results indicated that CAPS targeting a single site has a limited ability to resist T7 phage infection; therefore, CAPS needs further improvement.

### Designing CRISPR-Cas9 with gRNAs targeting two sites in the T7 phage genome can increase the defense ability of *E*. *coli* MG1655

**Figure 3:**
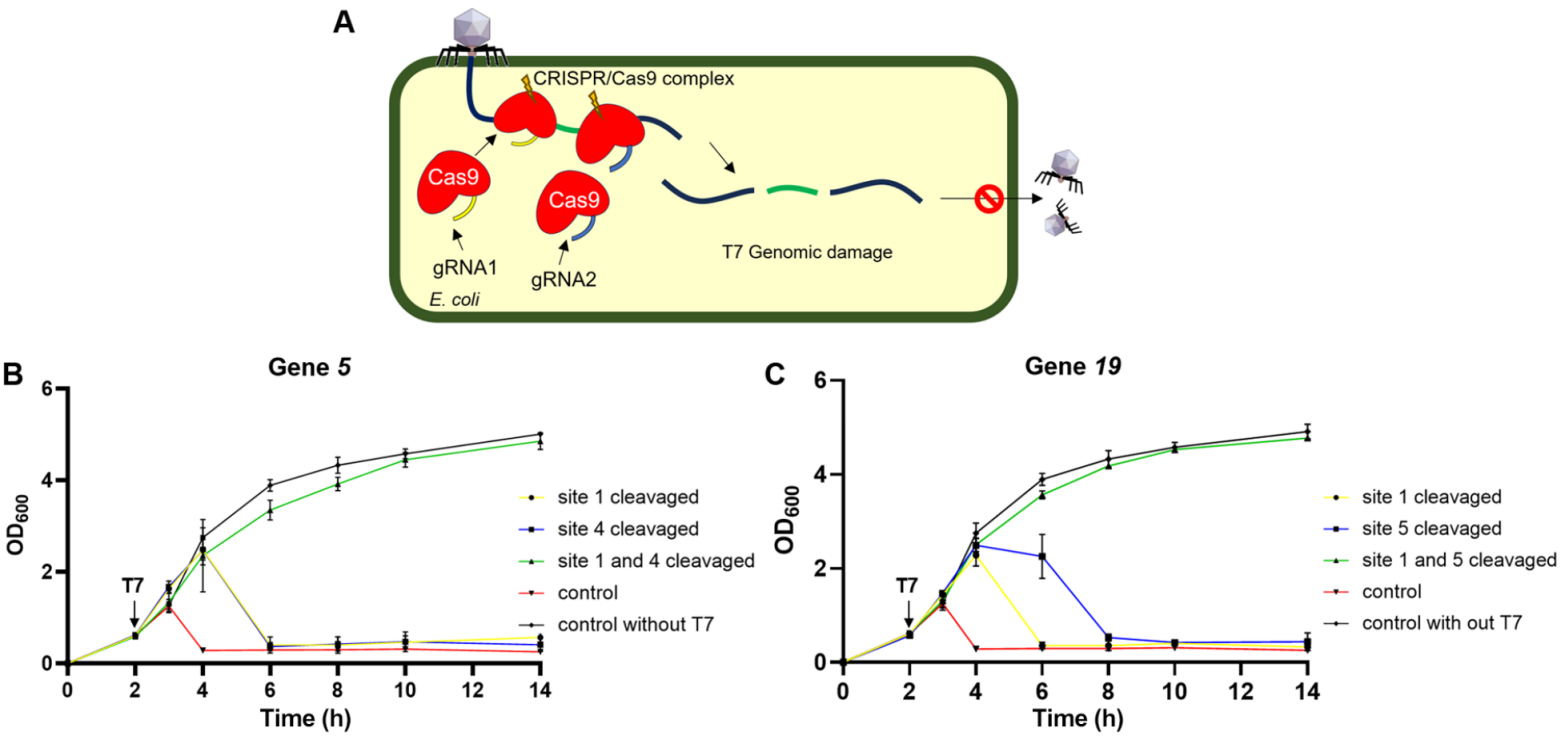
Figure 3 D-CAPS can enhance the defense ability of MG1655 (A) Schematic representation of the function of D-CAPS. (B) Growth curve showing that D-CAPS, which targets gene 5 of T7, can enable MG1655 to defend against T7 infection completely at an MOI of 0.02. (C) Growth curve showing that D-CAPS, which targets gene 19 of T7, can enable MG1655 to defend against T7 infection completely at an MOI of 0.02. The “control” represents the wild-type MG1655 with T7.

Since CAPS targeting a single site has limited effectiveness in resisting T7 phage infection, we attempted to improve CAPS by expressing multiple gRNAs in *E*. *coli* MG1655. By introducing various DSBs into the genome, we aimed to cause the phage to lose essential genes, thereby restricting infection ( Figure 3A). We attempted to create *E*. *coli* strains that simultaneously express two gRNAs that were designed previously, such as those targeting sites 1 and 2 in gene *19* of the T7 phage. However, the EOP of the T7 phage on these strains showed almost no difference from the lower EOP values of the two gRNAs ( Supplementary Figure S2). Thus, we speculate that the greater number of EOP sites may be resulted from poor cleavage efficiency at the targeted sequence, leading to negligible antiphage effects at those sites.

Thus, two sites with the lowest escape rate in each gene were selected and combined, namely, sites 1 and 4 for gene *5* and sites 1 and 5 for gene *19*. Two different plasmids expressing these two types of gRNAs were constructed. These two plasmids, together with pEcCas, form an improved CAPS called D-CAPS. Next, the D-CAPS were transformed into wild-type MG1655. These MG1655 strains with D-CAPS were then infected with different concentrations of the T7 phage. Fortunately, no plaques were observed on the double-layer plates ( Supplementary Figure S1), indicating that D-CAPS, which targets two sites of the T7 phage genome, successfully resisted T7 phage infection.

To confirm whether the presence of D-CAPS affects the normal growth of *E*. *coli* MG1655 and to observe the behavior of these MG1655 strains with D-CAPS when faced with T7 phage infection in a liquid environment, the growth curves of these strains infected with T7 phage at an MOI of 0.02 were subsequently plotted. The growth of these strains was not affected by the T7 phage and was very similar to the growth of the wild-type MG1655 at any stage of culture ( Figure 3B,C). Compared with the previous four MG1655 strains with CAPS, D-CAPS was better at eliminating the impact of T7 phage infection.

**Figure 4:**
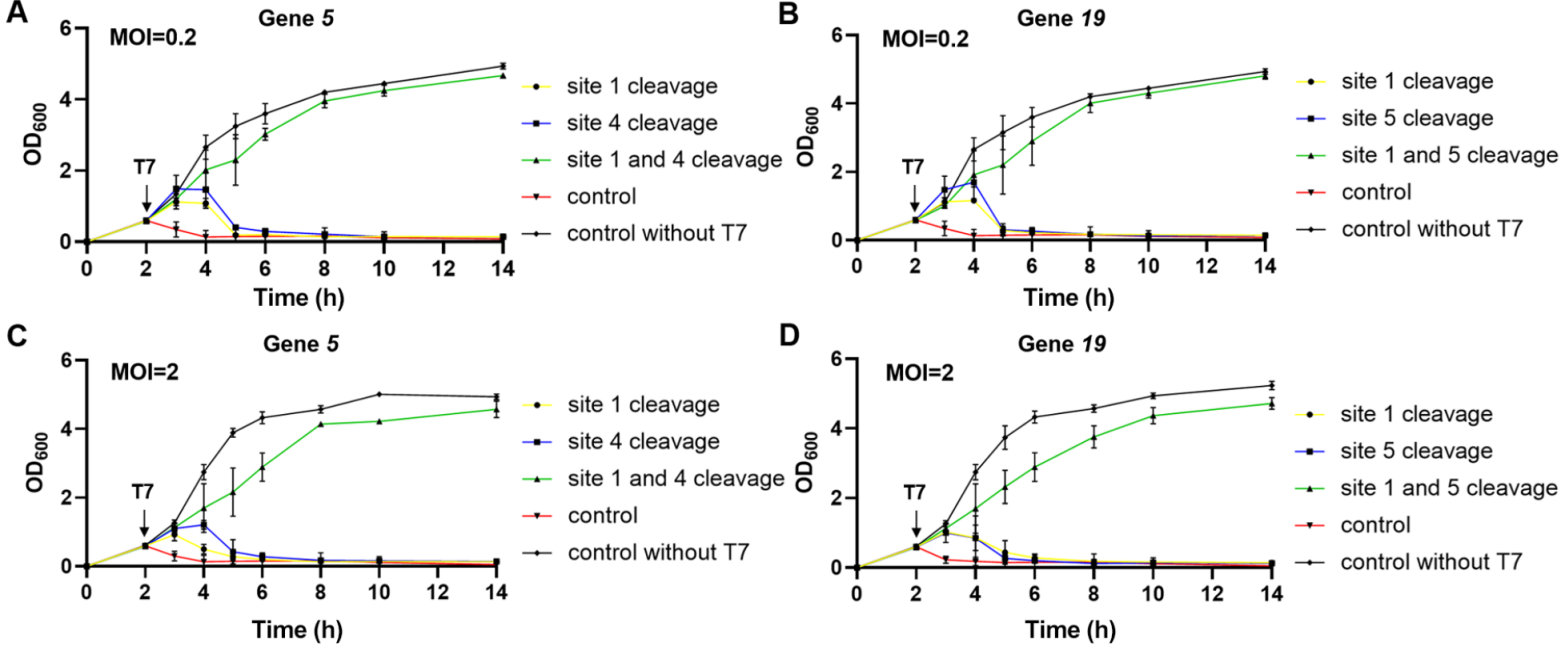
Figure 4 MG1655 with D-CAPS can defend against T7 at various concentrations (A) Growth curve demonstrating that D-CAPS, which targets gene 5 of T7, can enable MG1655 to defend against T7 infection completely at an MOI of 0.2. (B) Growth curve showing that D-CAPS, which targets gene 19 of T7, can enable MG1655 to defend against T7 infection completely at an MOI of 0.2. (C) Growth curve demonstrating that D-CAPS targeting gene 5 of T7 can enable MG1655 to defend against T7 infection completely at an MOI of 2. (D) Growth curve showing that D-CAPS, which targets gene 19 of T7, can enable MG1655 to defend against T7 infection completely at an MOI of 2. The “control” represents the wild-type MG1655 with T7.

We were subsequently curious about the performance of these MG1655 strains with D-CAPS in the face of more severe T7 phage infection. Therefore, the growth curves of these two MG1655 strains with D-CAPS when exposed to the T7 phage at MOIs of 0.2 and 2 were plotted. The results showed that even with a 10-fold or 100-fold increase in T7 concentration, these two MG1655 strains with D-CAPS still exhibited good anti-T7 effects. In the presence of a high concentration of T7, the wild-type MG1655 began to lyse within 1 h after the addition of T7. Lysis was observed in MG1655 with CAPS 2–3 h after phage addition, whereas MG1655 with D-CAPS continued to grow similarly to wild-type MG1655 even after 12 h ( Figure 4). Clearly, D-CAPS significantly enhances the anti-T7 capability of *E*. *coli*.

### D-CAPS is also effective in *E*. *coli* BL21 (DE3)

**Figure 5:**
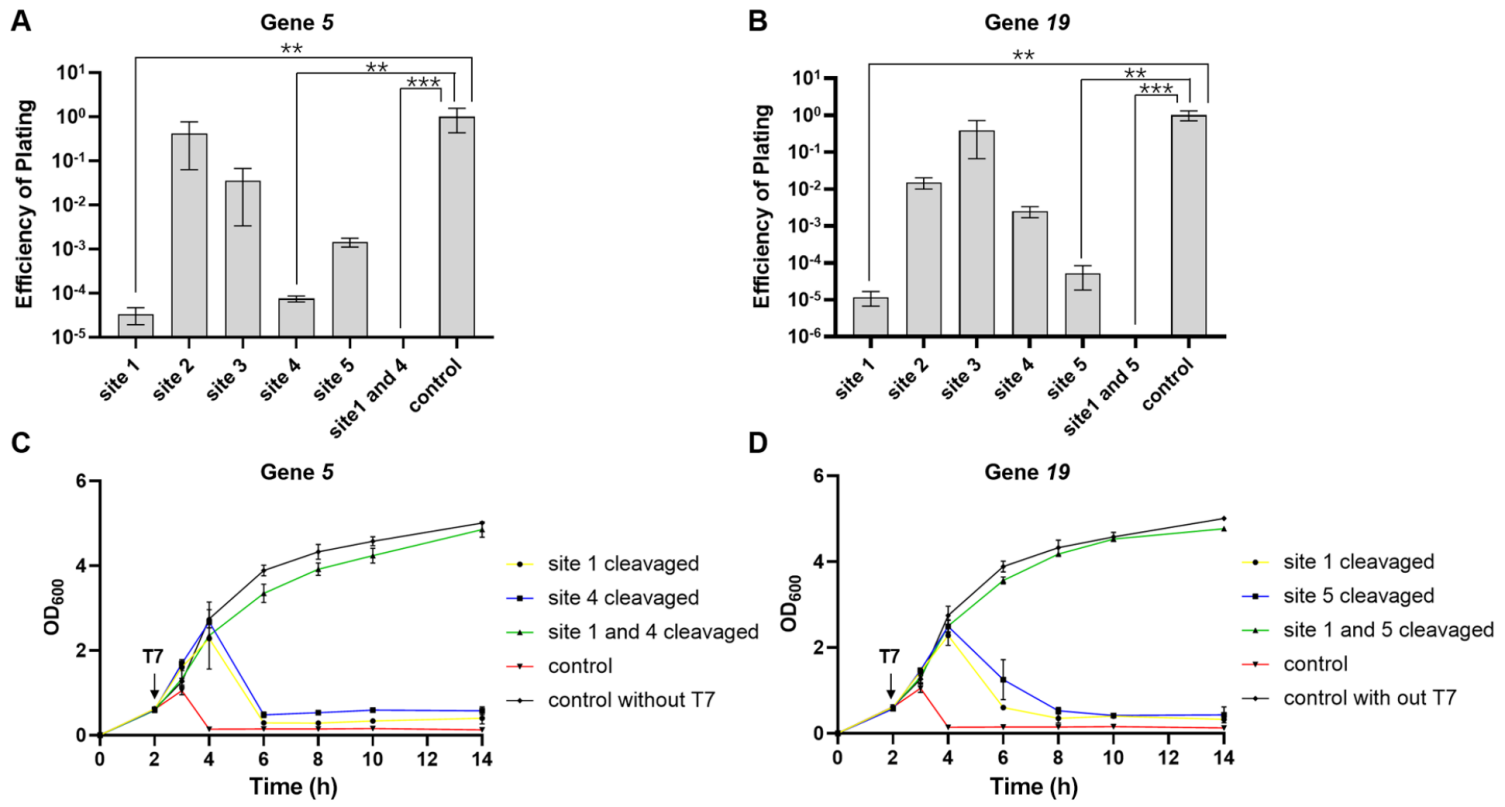
Figure 5 D-CAPS can be extended to BL21(DE3) and functions effectively (A) BL21(DE3) with D-CAPS targeting gene 5 of T7 completely defends against T7 infection. (B) BL21(DE3) with the D-CAPS-targeting gene 19 of T7 completely defends against T7 infection. (C) Growth curve showing that D-CAPS targeting gene 5 of T7 can enable BL21(DE3) to defend against T7 infection completely at an MOI of 0.02. (D) Growth curve showing that D-CAPS, which targets gene 19 of T7, can enable BL21(DE3) to defend against T7 infection completely at an MOI of 0.02; *P < 0.05; **P < 0.01; ***P < 0.001.

In addition to MG1655, many common industrial strains of *E*. *coli*, such as BL21(DE3), which are frequently used for expressing recombinant proteins in industry, are also susceptible to T7 phage contamination. Compared with MG1655, BL21(DE3) has an even weaker endogenous antiphage system [ 28, 30, 31]. Therefore, both the CAPS and the D-CAPS were introduced into BL21(DE3). These strains were then infected with different concentrations of T7 phage, and the EOP was calculated. The results showed that the EOP of the T7 phage in BL21(DE3) was not significantly different from that in MG1655 ( Figure 5A,B), demonstrating that D-CAPS is effective in other *E*. *coli* strains as well.

Furthermore, to determine whether the growth of BL21(DE3) with CAPS or D-CAPS differs from that of the wild-type and to evaluate its anti-T7 ability, the growth curves of these BL21(DE3) strains when exposed to T7 phage at an MOI of 0.02 were plotted. The growth of BL21(DE3) with D-CAPS was not significantly different from that of wild-type BL21(DE3). In contrast, BL21(DE3) with CAPS also failed to restrict T7 phage infection completely, resulting in significant lysis 2–4 h after T7 addition, whereas the wild-type MG1655 was almost completely lysed within 1–2 h ( Figure 5C,D). This confirms that our CRISPR-Cas9-based double-site phage defense system remains effective in other *E*. *coli* strains.

### Evaluating the protein expression ability of phage-resistant *E*. *coli* through GFP fluorescence expression

**Figure 6:**
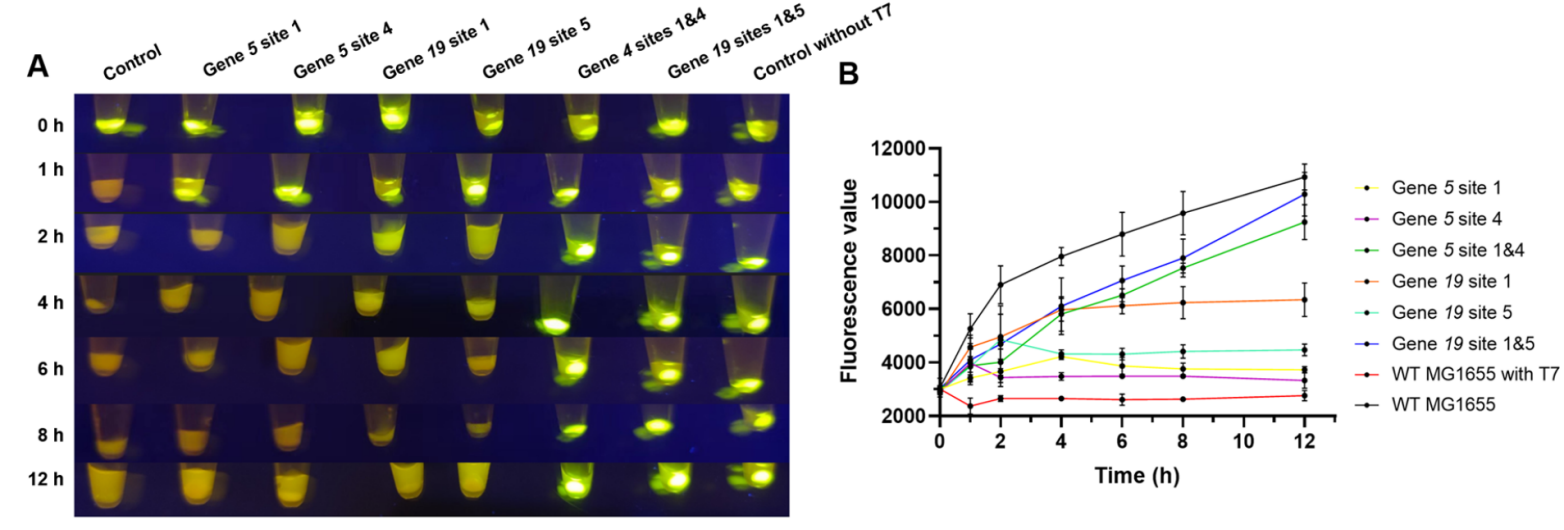
Figure 6 MG1655 carrying D-CAPS could normally express proteins even in the presence of phages (A) The fluorescence intensity observed in the pellet of centrifuged MG1655 cultures containing T7 phage demonstrated that MG1655 carrying D-CAPS exhibited a similar GFP expression ability to the wild-type control without T7 phage. (B) The protein expression ability of MG1655 carrying D-CAPS was evaluated via a fluorescence spectrophotometer.

To evaluate the protein expression capability of *E*. *coli* strains with the anti-T7 system, four MG1655 strains with CAPS and two MG1655 strains with D-CAPS were transformed with the plasmid pGFP, which constitutively expresses the fluorescent protein GFP. After the bacteria were cultured to the logarithmic growth phase, 3 μL of T7 phage stock solution (10 ^8^ PFU/μL) was added, and the cultures were subjected to measurements of fluorescence expression levels for each bacterial mixture at regular time points. The results revealed that the fluorescence in MG1655 with CAPS was almost undetectable 1–2 h after T7 addition, indicating that these *E*. *coli* strains had been lysed by T7 phage. Fortunately, the fluorescence intensity of the two *E*. *coli* strains with D-CAPS was similar to that of the wild-type MG1655 without T7. When T7 was added to wild-type MG1655, fluorescence was no longer observable after 1 h of culture ( Figure 6A). Additionally, we plotted the fluorescence intensity curves for the strains mentioned above, periodically quantifying the fluorescence expression levels of each culture. Two hours after T7 addition, the fluorescence intensity of the MG1655 strain carrying CAPS remained nearly unchanged, indicating that these *E*. *coli* cells had been lysed by T7 and no longer expressed GFP. In contrast, the fluorescence intensity of the two MG1655 strains carrying D-CAPS continued to increase over time, with levels similar to those of the wild-type MG1655 carrying pGFP. On the other hand, the fluorescence intensity of the wild-type MG1655 exposed to the T7 phage began to decrease significantly after 1 h of incubation and remained low ( Figure 6B). Therefore, the CRISPR-Cas9-based anti-T7 system provides T7 phage resistance to the host *E*. *coli* without affecting protein expression in *E*. *coli*.

## Discussion

As an important industrial chassis cell, *E*. *coli* plays an important role in the field of synthetic biology [10]. However, contamination by bacteriophages, such as T7 phage, often severely disrupts the industrial production of *E*. *coli*. Many studies have reported the elimination of the impact of phage contamination, with the introduction of exogenous antiphage systems into fermentation strains being the most favored approach [ 25– 27]. In recent years, with the rise of CRISPR-Cas9 technology, researchers have attempted to express the CRISPR-Cas9 system in *E*. *coli* to achieve immunity against T7 phage [28]. However, its anti-T7 efficacy requires improvement, as the system cannot completely resist T7 phage infection but only delays the lysis of the host bacteria [28]. Additionally, it relies on expensive inducers to initiate the expression of the antiphage system.

In this study, an efficient CRISPR-Cas9-based antiphage system named CAPS, which is suitable for *E*. *coli*, was constructed. To ensure high cleavage efficiency, we first analyzed the growth stages of the T7 phage, and two essential genes, *5* and *19*, involved in genome replication and assembly, respectively, were selected. Five sites for each gene were chosen for targeting, and the EOP of the T7 phage at these targeted sites was tested. Similar to previous studies, these *E*. *coli* strains with CAPS could not resist T7 phage infection completely. We hypothesized that T7 phage might escape Cas9 cleavage through synonymous mutations on the basis of our previous research [24]. To increase the resistance of MG1655 strains to T7 phage, we next optimized the system by combining the two sites with the highest cleavage efficiency in each targeted gene, and an improved D-CAPS system was constructed. In both double-layer plates and liquid culture environments, we demonstrated that the MG1655 strain with D-CAPS can resist infection by T7 phage at various concentrations.

Furthermore, we extended this system to the BL21 (DE3) strain and obtained similar results, confirming that our D-CAPS is versatile and applicable to multiple *E*. *coli* strains. Finally, the protein expression capability of these MG1655 strains with D-CAPS was evaluated using a fluorescent protein. The results showed that these MG1655 strains with D-CAPS could normally express the target protein in the presence of the T7 phage, with a fluorescence intensity similar to that of wild-type *E*. *coli*.

Our D-CAPS has a range of advantages or innovations compared with the previously reported CRISPR-Cas9-based *E*. *coli* phage defense system. First, D-CAPS achieves complete defense against T7 phage through the strategic selection of effective cleavage sites, which is more effective than other CRISPR-Cas9-based antiphage systems [28]. Genes *5* and *19* are directly involved in genome replication, repair, and maturation processes in the T7 phage. Therefore, targeting these sites, compared with other sites, such as structural protein genes, can significantly reduce the probability of correct genome repair [32]. Furthermore, we demonstrated that the dual-site targeting approach of CRISPR-Cas9 requires two efficient cleavage sites. Therefore, it is essential to test cleavage efficiency beforehand to select the two most efficient sites for optimal defense ( Supplementary Figure S2). However, screening the efficiency of multiple cleavage sites is a labor-intensive task. To establish an efficient screening process, bioinformatics tools and algorithms such as CRISPOR, CasOFFinder, Off-Spotter, and sgRNA Designer can be used to predict potential high-efficiency cleavage sites in advance [ 33– 36]. By combining these predictive algorithms with experimental validation, the design cycle can be shortened, reducing the time required to identify effective cleavage sites. Second, on the basis of the prediction of cleavage site efficiency in the previous section, the construction process of this system was short; it took only 4 days from primer design to obtain antiphage *E*. *coli*, and it did not require any inducer or strain rotation strategies. In the double-layer plate experiments, strong inhibitory effects against various concentrations of T7 phage were exhibited by our anti-T7 *E*. *coli* ( Supplementary Figure S1). Finally, the *E*. *coli* strain with D-CAPS we constructed could still grow normally even in a high-concentration phage environment at an MOI of 2, and its growth situation was similar to that of the wild-type. In contrast, previous studies have shown that anti-T7 *E*. *coli* BL21(DE3) began to lyse 2–4 h after phage addition, whereas the few surviving BL21(DE3) strains that were resistant to T7 successfully exhibited poor growth, with an OD _600_ value of only approximately 2. In this study, BL21(DE3) with D-CAPS presented OD _600_ values reaching 4 under the same culture conditions ( Figure 4C,D).

In addition, there is still potential for further optimization of this system. For example, the gRNA plasmid can be replaced by a gRNA array to target more sites, thereby achieving resistance to multiple kinds of phages. On the other hand, integrating this system into the host *E*. *coli* genome could reduce the use of antibiotics during production. Considering the potential off-target risks of CRISPR-Cas9 and phage mutation issues in long-term cultures, it is advisable to use various bioinformatics tools to evaluate and screen the cleavage efficiency of gRNAs in advance to reduce the likelihood of phage mutations [37]. Factors such as the sequence composition of the host organism’s genome, the secondary structure of the target DNA, and proximity to specific motifs are considered in these tools, thus ensuring the high efficiency of gRNAs while effectively minimizing off-target risks.

In summary, we developed an efficient CRISPR-Cas9-based anti-T7 system and transformed it into two *E*. *coli* strains, MG1655 and BL21(DE3). These strains exhibited high resistance to T7 phage and became safer and more resilient protein expression hosts. This study not only enhances the safety and sustainability of fermentation but also advances microbial production technology, laying a foundation and providing a reference for the development of synthetic biology techniques.

## Supporting information

703FigS1-2TabS1-2

## Supplementary Data

Supplementary data is available at *Acta Biochimica et Biphysica Sinica* online.
