# Supplementary material for: D-CAPS: an efficient CRISPR-Cas9-based phage defense system for E. coli : A novel strategy for E. coli to resist T7 based on CRISPR-Cas9
Source: Acta Biochim Biophys Sin (Shanghai). 2025 Apr 28;57(8):1244–51. doi: 10.3724/abbs.2024208 (PMC12368531; doi:10.3724/abbs.2024208)
Supplement: 703FigS1-2TabS1-2 [file 703FigS1-2TabS1-2.docx]

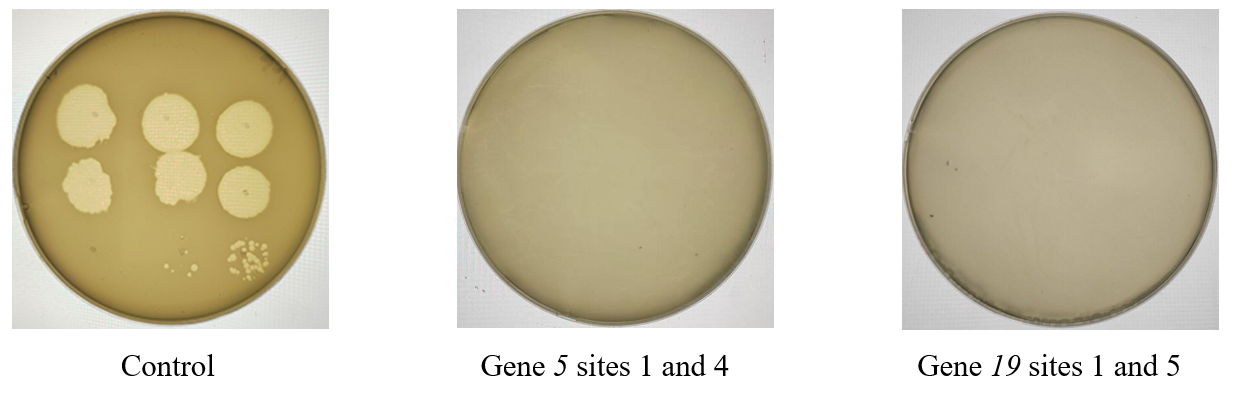


**Supplemental Figure** **S1.** **Efficient multilocus cleavage can effectively help *E. coli* resist the infection of T7 phage**


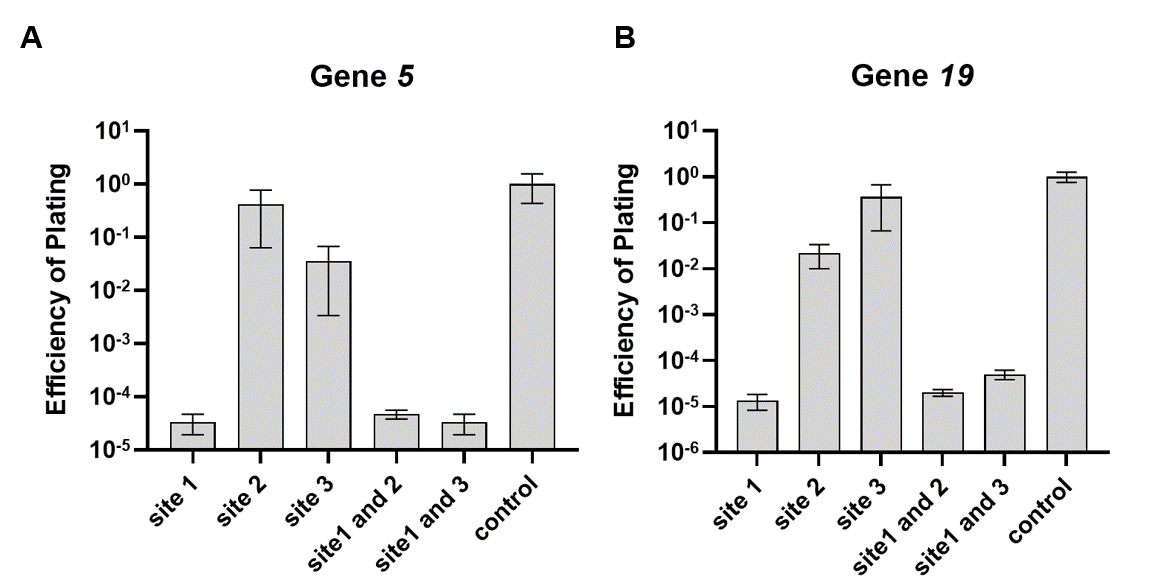


**Supplemental Figure S2. The EOP of T7 phage on strains targeting two sites with significantly different cleavage efficiencies is almost the same as the lower EOP value of the two gRNAs** (A) Comparison of the results of multi-locus cleavage and single-site cleavage of gene *5*. (B) Comparison of the results of multi-locus cleavage and single-site cleavage of gene *19*.

**Supplementary Table S1. Plasmids and strains used in this study**

| Strains or plasmids | Description | Source |
| --- | --- | --- |
| Strains |  |  |
| *E. coli* DH5α | Commercial transformation host | Gibco BRL, Life Technologies |
| *E. coli* MG1655 | Host for testing the efficiency of plating | Lab storage |
| *E. coli* BL21(DE3) | Host for testing the efficiency of plating | Lab storage |
| Bacteriophage T7 | Phage for testing the efficiency of plating | Lab storage |
| Plasmids |  |  |
| pEcCas | Constitutive expression of Cas9 and inducible expression of λ-Red recombination systems | Lab storage |
| pEcgRNA-T7-5.1 | Derived from pEcgRNA, target T7 *5* site 1 in Bacteriophage T7 | This study |
| pEcgRNA-T7-5.2 | Derived from pEcgRNA, target T7 *5* site 2 in Bacteriophage T7 | This study |
| pEcgRNA-T7-5.3 | Derived from pEcgRNA, target T7 *5* site 3 in Bacteriophage T7 | This study |
| pEcgRNA-T7-5.4 | Derived from pEcgRNA, target T7 *5* site 4 in Bacteriophage T7 | This study |
| pEcgRNA-T7-5.5 | Derived from pEcgRNA, target T7 *5* site 3 in Bacteriophage T7 | This study |
| pEcgRNA-T7-19.1 | Derived from pEcgRNA, target T7 *19* site 1 in Bacteriophage T7 | This study |
| pEcgRNA-T7-19.2 | Derived from pEcgRNA, target T7 *19* site 2 in Bacteriophage T7 | This study |
| pEcgRNA-T7-19.3 | Derived from pEcgRNA, target T7 *19* site 3 in Bacteriophage T7 | This study |
| pEcgRNA-T7-19.4 | Derived from pEcgRNA, target T7 *19* site 4 in Bacteriophage T7 | This study |
| pEcgRNA-T7-19.5 | Derived from pEcgRNA, target T7 *19* site 5 in Bacteriophage T7 | This study |
| pEcgRNA-T7-5.1-Cm | Target T7 *5* site 1 in T7 and replaced the selection marker with chloramphenicol resistance | This study |
| pEcgRNA-T7-19.1-Cm | Target T7 *19* site 1 in T7 and replaced the selection marker with chloramphenicol resistance | This study |
| pGFP | Constitutive expression of GFP to evaluate protein expression levels | Lab storage |

**Supplementary Table S2.** **Oligonucleotides used in this study**

| **Oligos** | **Sequence (5’→3’)** |
| --- | --- |
| T7-5.1-gRNA-up | tcctaggtataatactagtAATCAAACGTGACAACACAAgttttagagctagaaatagc |
| T7-5.2-gRNA-up | tcctaggtataatactagtGGCCACACAGTCACGCGCTCgttttagagctagaaatagc |
| T7-5.3-gRNA-up | tcctaggtataatactagtGGCCACACAGTCACGCGCTCgttttagagctagaaatagc |
| T7-5.4-gRNA-up | tcctaggtataatactagtGGCCACACAGTCACGCGCTCgttttagagctagaaatagc |
| T7-5.5-gRNA-up | tcctaggtataatactagtGGCCACACAGTCACGCGCTCgttttagagctagaaatagc |
| T7-19.1-gRNA-up | tcctaggtataatactagtTGAGTACGATGAGAACCCTGgttttagagctagaaatagc |
| T7-19.2-gRNA-up | tcctaggtataatactagtAGGAGTTGATGTGTACTCTGgttttagagctagaaatagc |
| T7-19.3-gRNA-up | tcctaggtataatactagtATGCCGAGAAGTACCCGCTGgttttagagctagaaatagc |
| T7-19.4-gRNA-up | tcctaggtataatactagtGCTGTGCTGTACACACTGAAgttttagagctagaaatagc |
| T7-19.5-gRNA-up | tcctaggtataatactagtCATCACGGTCAAAGCGCACTgttttagagctagaaatagc |
| gRNA-dn-univers | ctagtattatacctaggactgagct |

Sequences in uppercase represent the guide sequence of gRNA used in gene cutting plasmids construction.
